# Are the results from a multiplex proteomic assay and a conventional immunoassay for NT-proBNP and GDF-15 comparable?

**DOI:** 10.1186/s12014-023-09393-1

**Published:** 2023-01-24

**Authors:** Emma Skau, Philippe Wagner, Jerzy Leppert, Johan Ärnlöv, Pär Hedberg

**Affiliations:** 1 Centre for Clinical Research, Västmanland County Hospital, Uppsala University, SE-72 189 Västerås, Sweden; 2 Department of Cardiology, Danderyd University Hospital, Stockholm, Sweden; 3 School of Health and Social Studies, Dalarna University, Falun, Sweden; 4 Division of Family Medicine and Primary Care, Department of Neurobiology, Care Sciences and Society (NVS), Karolinska Institutet, Huddinge, Sweden; 5 Department of Clinical Physiology, Västmanland County Hospital, Västerås, Sweden

**Keywords:** Biomarkers, Proximity extension assay, Proteomic, N-terminal pro-brain natriuretic peptide, Growth differentiation factor 15, Immunoassay, Peripheral arterial disease

## Abstract

**Background:**

We aimed to compare absolute plasma concentrations of N-terminal pro-brain natriuretic peptide (NT-proBNP) and growth differentiation factor 15 (GDF-15) obtained by a conventional immunoassay with the corresponding relative concentrations from a proximity extension assay (PEA) and compare the prognostic impact of the protein levels obtained from these assays.

**Methods:**

We evaluated 437 patients with peripheral arterial disease (PAD) and a population-based cohort of 643 individuals without PAD. Correlations were calculated using Spearman’s rank correlation coefficients (rho). The discriminatory accuracy of the protein levels to predict future cardiovascular events was analyzed with Cox regression and presented as time-dependent areas under the receiver-operator-characteristic curves (tdAUCs).

**Results:**

For NT-proBNP, the two assays correlated with rho 0.93 and 0.93 in the respective cohort. The PEA values leveled off at higher values in both cohorts. The corresponding correlations for GDF-15 were 0.91 and 0.89. At 5 years follow-up, the tdAUCs in the patient cohort were similar for NT-proBNP and GDF-15 regardless of assay used (0.65–0.66). The corresponding tdAUCs in the population-based cohort were between 0.72 and 0.77.

**Conclusion:**

Except for the highest levels of NT-proBNP, we suggest that PEA data for NT-proBNP and GDF-15 reliably reflects absolute plasma levels and contains similar prognostic information.

## Introduction

Tracking plasma proteins as biomarkers for diagnosis, prediction and treatment in different disease areas has developed over the past decades. The biomarker research field has expanded due to advancements in analysis technologies. Novel multiplex protein biomarker assays have opened the door to new dimensions in human humoral biomarker research. Such assays may help study disease mechanisms and discover novel clinical biomarkers for diagnosis, prognosis, and treatment response through untargeted analyses of a large number of candidate proteins [1–3].

The multiplex high-throughput proximity extension assay (PEA), by Olink Bioscience, Uppsala, Sweden, is based on proximity-dependent DNA polymerization. This technique enables the simultaneous detection of multiple proteins with a minimal sample volume (one microliter of plasma). The PEA is highly sensitive and specific for detecting the target-specific proteins due to the dual and proximal binding of the PEA probes [4]. There has been an exponential increase of research articles presenting clinical data from this PEA technology (http://www.olink.com). Many promising PEA-based biomarkers have thereby been identified, including in the field of cardiovascular research [4–9]. Despite this, few of these biomarkers are translated into clinical practice. This slow transition might partly be because PEA is designed to quantify relative protein concentrations and not the absolute concentration values usually used in clinical practice. Independent information on how well the protein biomarker levels assessed with a PEA platform correlate with absolute concentration levels from established assays are scarce [10].

N-terminal pro-brain natriuretic peptide (NT-proBNP) and growth differentiation factor 15 (GDF-15) are well-studied prognostic cardiovascular (CV) biomarkers. They are both included in the PEA platform Proseek Multiplex CVD I 96 × 96 panel (Olink Bioscience, Uppsala, Sweden) [11–17], as well as available in established conventional commercially available immunoassays.

We aimed to evaluate how the relative plasma concentrations of NT-proBNP and GDF-15 obtained with the PEA correlate with the absolute plasma concentrations obtained with a conventional assay in individuals with and without verified manifest peripheral arterial disease (PAD). Further, we aimed to compare the plasma values of the proteins obtained from these two techniques to predict CV events.

## Method

### Study populations

This study included two cohorts, participants from the Peripheral Artery Disease in Västmanland study (PADVa), and a population-based sample recruited as control subjects for the Västmanland Myocardial Infarction Study (VaMIS); ClinicalTrials.gov number, NCT01452178). Individuals with a verified PAD diagnosis in the population-based cohort were excluded.

#### The PADVa cohort

Consecutive patients referred to the Vascular Ultrasound Laboratory of the Department of Vascular Surgery, Västmanland County Hospital, Västerås, Sweden, were evaluated for inclusion between April 2006 and February 2011. At least one of the following criteria were required to be enrolled in the study:

(i) symptoms of claudication intermittent (described as pain from lower limb reproduced by exercise and relieved within 10 min’ rest) in combination with ankle-brachial index (ABI) ≤ 0.9;
(ii) symptoms of claudication intermittent with signs of occlusive arterial disease on ultrasound examination in the ipsilateral extremity;
(iii) ultrasound verified stenosis or occlusion of the internal carotid artery (ICA).

Among 614 patients fulfilling the criteria for participation, 162 (26.4%) rejected inclusion, and 15 (2.4%) were excluded due to any missing biomarker values, leaving 437 individuals with carotid or lower limb artery disease included in the study.

#### The population-based cohort

In the VaMIS study, consecutive patients hospitalized for acute myocardial infarction from November 2005 to May 2011 were included [18]. Using the Swedish Population register, a control subject was recruited from the general population for each included VaMIS patient. A random individual with the closest date of birth, same sex, and same municipality as an included VaMIS patient was invited to participate. The patients from the PADVa cohort and controls in the VaMIS study underwent the same study protocol [15]. From the 855 control subjects in the VaMIS study, 175 individuals (20.5%) were excluded as they fulfilled the criteria for PAD specified above. Further, 37 (4.3%) with missing biomarker values were excluded, leaving 643 subjects for analysis in the population-based cohort.

### Study protocol

The study protocol for both cohorts included medical history, current medication and smoking habits obtained from self-administered questionnaires. Diagnoses of previous CV events and diabetes mellitus and were confirmed from medical records. Hypertension was defined if diagnosed by a physician and prescribed antihypertensive medications. The ABI was calculated as the highest of the systolic blood pressures in the dorsalis pedis artery and the posterior tibial artery divided by the systolic blood pressure in the arm. ABI was defined as abnormal if ≤ 0.90 or 1.40 in any leg. Stenosis in the internal carotid artery (ICA) was considered if the ultrasonography investigation detected a localized protrusion of the vessel wall into the lumen in combination with turbulent color Doppler flow, spectral broadening in the spectral Doppler flow, and a peak systolic flow velocity of ≥ 1.2 m/s [19]. Left ventricular ejection fraction (LVEF) was assessed using the biplane Simpson’s formula [20]. In subjects for whom it was impossible to obtain the Simpson’s LVEF, a visual estimation of LVEF was made.

### Blood samples

All the blood samples were taken after a night fasting by trained staff and immediately sent to the accredited Laboratory of Clinical Chemistry, Västmanland County Hospital, Västerås, Sweden, for analyses or freezing. Blood samples aimed for freezing were obtained in 5 ml lithium heparin-coated vacuum tubes. The tubes were centrifuged at 2000 g for 10 min (Becton Dickinson and Co., Franklin Lakes, New Jersey, USA) or 2200 g for 10 min (Vacuette, Greiner Bio-One GmbH, Austria). The plasma was reallocated to 5 ml plastic tubes, frozen and stored at − 70 °C within 2 h.

#### Blood analyses at baseline

Plasma levels of NT-proBNP were determined at baseline by a sandwich immunoassay using monoclonal antibodies and biotin-streptavidin separation (Elecys 1010 and Cobas e411 instruments, Roche Diagnostics, Germany) [21]. The analytical range was 5–35,000 ng/L and total coefficient of variation (CVa) of 5.4% and 4.4% at 101 ng/L and 908 ng/L, respectively. Serum creatinine was measured by the Jaffé method standardized against isotope dilution mass spectrometry (Synchron LX 20 and UniCel DxC instruments, Beckman Coulter, USA) with a total CV of 5.3% and 2.4% at 90 µkat/L and 379 µkat/L, respectively. Total cholesterol was measured by enzymatic conversion of cholesterolesterase by a time-endpoint method (Synchron LX 20 and UniCel DxC instruments, Beckman Coulter, USA) with a total CV of 1.5% and 1.6% at 3.18 mmol/L and 7.7 mmol/L, respectively. HbA1c was measured using high-performance liquid chromatography using cation exchange separation and calibrated against the Swedish Mono S method (TOSOH automated Glycohemoglobin Analyzer G7, Tosoh, Japan). HbA1c Mono S was converted to IFCC units by the equation $HbA1c\left(IFCC\right)=HbA1c\left(Mono S\right)\times 10.45-10.62$. This formula differs slightly from the IFCC master equation $HbA1c\left(IFCC\right)=HbA1c\left(Mono S\right)\times 10.11-8.94$ and is due to a recalculation in 2004 [22, 23].

#### Analyses on thawed blood samples

Before analysis, the samples were thawed at room temperature, mixed and centrifuged at 3470 g at 4 °C for 15 min and aliquoted into a microtiter plate using a pipetting robot, the Tecan Freedom Evolyze. The analyses were performed at the Clinical Biomarkers Facility, Science for Life Laboratory, Uppsala University, Uppsala, Sweden.

Plasma levels of GDF-15 were analysed in 2017 by a an immunoassay based on the specific Elecsys electrochemiluminescence (ECL) detection system (Roche Diagnostics) [24]. The analytical range was 400–20,000 ng/L with a total CVa of 4.9%.

The Proseek Multiplex CVD I 96 × 96 panel (Olink Bioscience, Uppsala, Sweden) was used to analyze 92 proteins previously associated with CVD or inflammation, including NT-proBNP and GDF-15 [16]. The content of the proteins in each plasma specimen was measured simultaneously by the binding of paired oligonucleotide-labeled antibodies to the target proteins. The subsequent formation of new polymerase chain reaction (PCR) amplification targets was detected and quantified by high-throughput real-time PCR. The measures are specified and presented in relative units on a binary logarithmic scale, the Normalized Protein Expression (NPX).

Validation of the assay including 90 proteins and seven samples analysed in nine separate runs showed a mean intra-assay coefficient of variation of 8% (range 4–13%) and an inter-assay coefficient of variation of 15% (range 11–39%).

### Follow-up and outcomes

The participants in both cohorts were followed from the index examination until an endpoint or, at the latest, December 31, 2013. The endpoint was a CV event defined as CV death (International Statistical Classification of Diseases and Related Health Problems, 10th revision code I00-I99) or hospitalization for myocardial infarction (MI) (I21), heart failure (I50, I11.0, or I25.5), or ischemic stroke (I63). Follow-up data were collected from the Swedish National Inpatient Register and the Swedish National Cause of Death Register.

### Statistical analyses

We present continuous variables as mean and standard deviation (SD) or median and interquartile range, and categorical variables as frequency and percentage.

Comparisons between the plasma values of NT-proBNP and GDF-15 measured by the conventional immunoassays and the PEA were performed using Spearman’s rank correlation coefficients (rho) with corresponding 95% confidence intervals (CI) and visualized in scatter plots with local polynomial regression lines (LOESS). Due to highly positively skewed plasma levels in the conventional analyses, the values were binary logarithmized (to the same scale as the PEA NPX values). To adjust for technical variation with internal controls in the PEA panels, the PEA and conventional plasma values were standardized to a mean of 0 and an SD of 1, grouped by the PEA panel.

Unadjusted Cox proportional hazard regression was used to evaluate the ability of the proteins to predict future CV events. The predictive values of the plasma levels of NT-proBNP and GDF-15 obtained with the PEA were compared with prediction based of plasma levels of the conventional assays in both cohorts. The unadjusted Cox regression results are presented as hazard ratios, with corresponding 95% CI. The discriminatory accuracy is presented as the time dependent areas under the receiver operating characteristics curves (tdAUC) [25]. The AUC values were calculated at 5 years of follow-up and demonstrated in plots.

Two-sided p-values < 0.05 were considered statistically significant. R (R Foundation for Statistical Computing; 2016, Vienna, Austria; https://www.r-project.org) were used for the statistical analyses.

## Results

### Patient characteristics

Baseline characteristics for each cohort are presented in Table 1. The mean age in the PADVa cohort was 70.0 years (SD 7.2 years) and 66.2 years (SD 9.5 years) in the population-based cohort. Females constituted 41.0% of the PADVa cohort and 29.9% of the population-based cohort. Compared with the population-based cohort, participants in the PADVa cohort had more comorbidity as hypertension, diabetes mellitus, previous MI and stroke, The median values for NT-proBNP and GDF-15 obtained from the two different analyze-techniques in the two different cohorts are presented in Table 1.

**Table 1:** Baseline characteristics of the PADVa cohort and population-based cohort

	PADVa n = 437	Population-based n = 643
Age (years)	70.0 ± 7.2	66.2 ± 9.5
Female sex	179 (41.0%)	192 (29.9%)
Ever smoked	331 (75.9%)	350(54.4%)
Body mass index (kg/m2)	27.1 ± 4.2	26.6 ± 3.6
Hypertension	336 (77.4%)	226 (35.1%)
Diabetes mellitus	110 (25.2%)	45 (7.0%)
Myocardial infarction	80 (18.3%)	21 (3.2%)
TIA/Stroke	44 (10.1%)	25 (3.9%)
Total cholesterol (mmol/L)	4.6 ± 1.2	5.6 ± 1.1
Creatinine (umol/L)	81 (69–97)	79 (69–90)
HbA1c (mmol/mol)	42.5 ± 10.7	37.9 ± 7.1
ICA stenosis		–
No stenosis	22 (0.5%)	–
30–50% stenosis	196 (44.9%)	–
50–70% stenosis	70 (16.0%)	–
> 70% stenosis/occlusion	62 (14.2%)	–
Abnormal ABI	255 (58.4%)	–
LVEF < 45%	24 (5.5%)	11 (1.7%)
Medication		
ACE-I	153 (35.1%)	73 (11.4%)
Statin	354 (81.2%)	111 (17.3%)
Aspirin	338 (77.5%)	117 (18.2%)
Betablocker	224 (51.4%)	130 (20.2%)
GDF-15 (NPX)	10.1 (9.6–10.6)	9.5(9.1–10.0)
GDF-15 (ng/L)	1379 (979–2083)	1027 (762–1377)
NT-proBNP (NPX)	5.1 (4.0–6.0)	3.7 (2.8–4.6)
NT-proBNP (ng/L)	169 (89–345)	77 (40–148.5)

Values are mean ± standard deviation, median (25th percentile-75th percentile), or frequency (percentage)

### Correlation between PEA versus conventional immunoassay for NT-proBNP

**Fig. 1:**
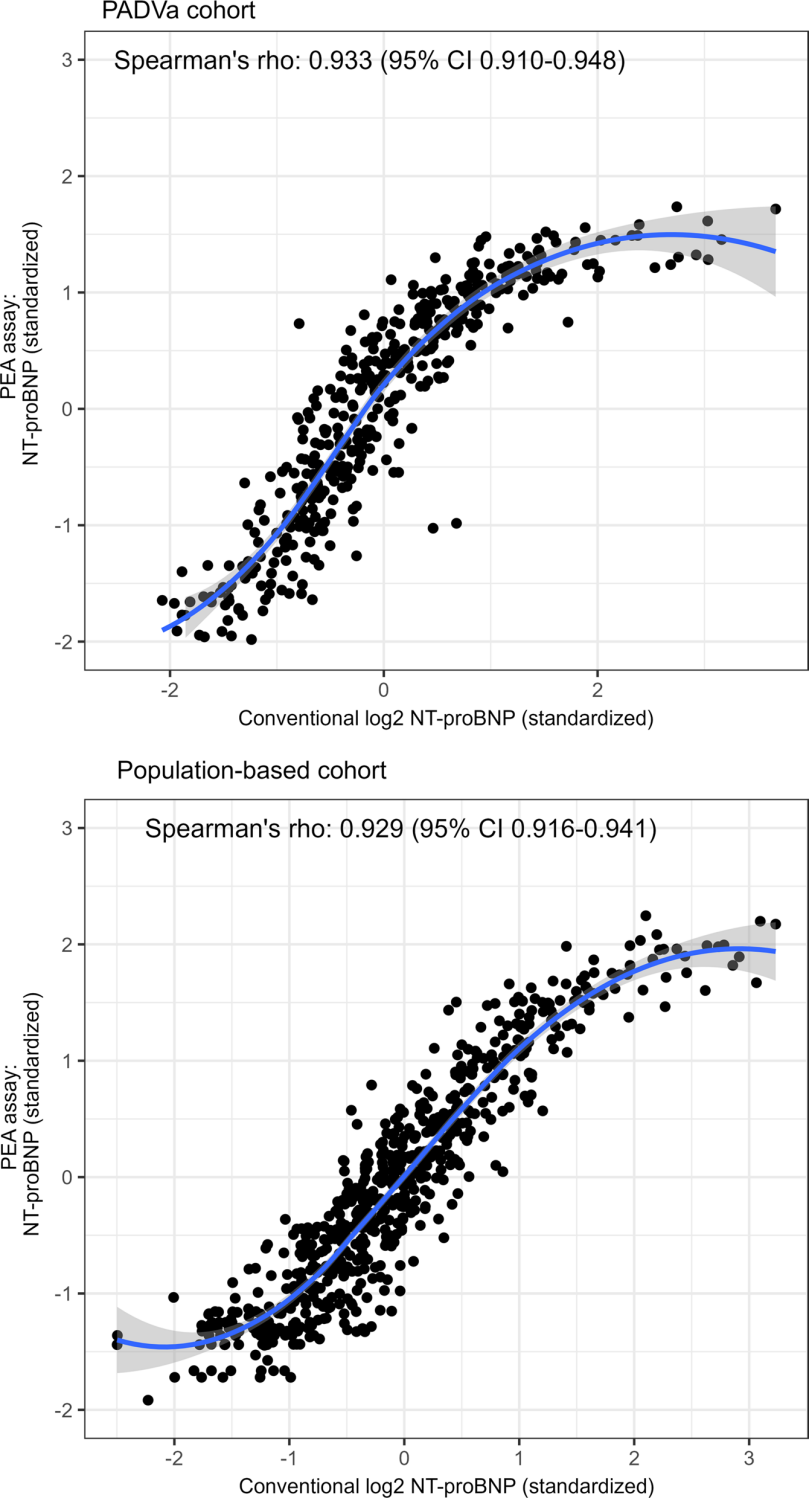
Scatter plots with corresponding curves constructed by LOESS (blue lines) for NT-proBNP analyzed with a conventional assay and a PEA assay in the studied cohorts. *LOESS* local polynominal regression lines, *NT-proBNP* N-terminal pro-brain natriuretic peptide, *PEA* proximity extension assay, *Spearman´s rho* Spearman’s rank correlation coefficient, *CI* confidence interval

The associations of plasma levels of NT-proBNP analyzed with the PEA and with the conventional immunoassay in both cohorts are presented in a scatter plot (Fig. 1). The correlation coefficients were 0.933 (95% CI 0.910–0.948) in the PADVa cohort and 0.929 (95% CI 0.916–0.941) in the population-based group. At higher levels of NT-proBNP the PEA values leveled off in both cohorts.

### Correlation PEA versus conventional immunoassay for GDF15

**Fig. 2:**
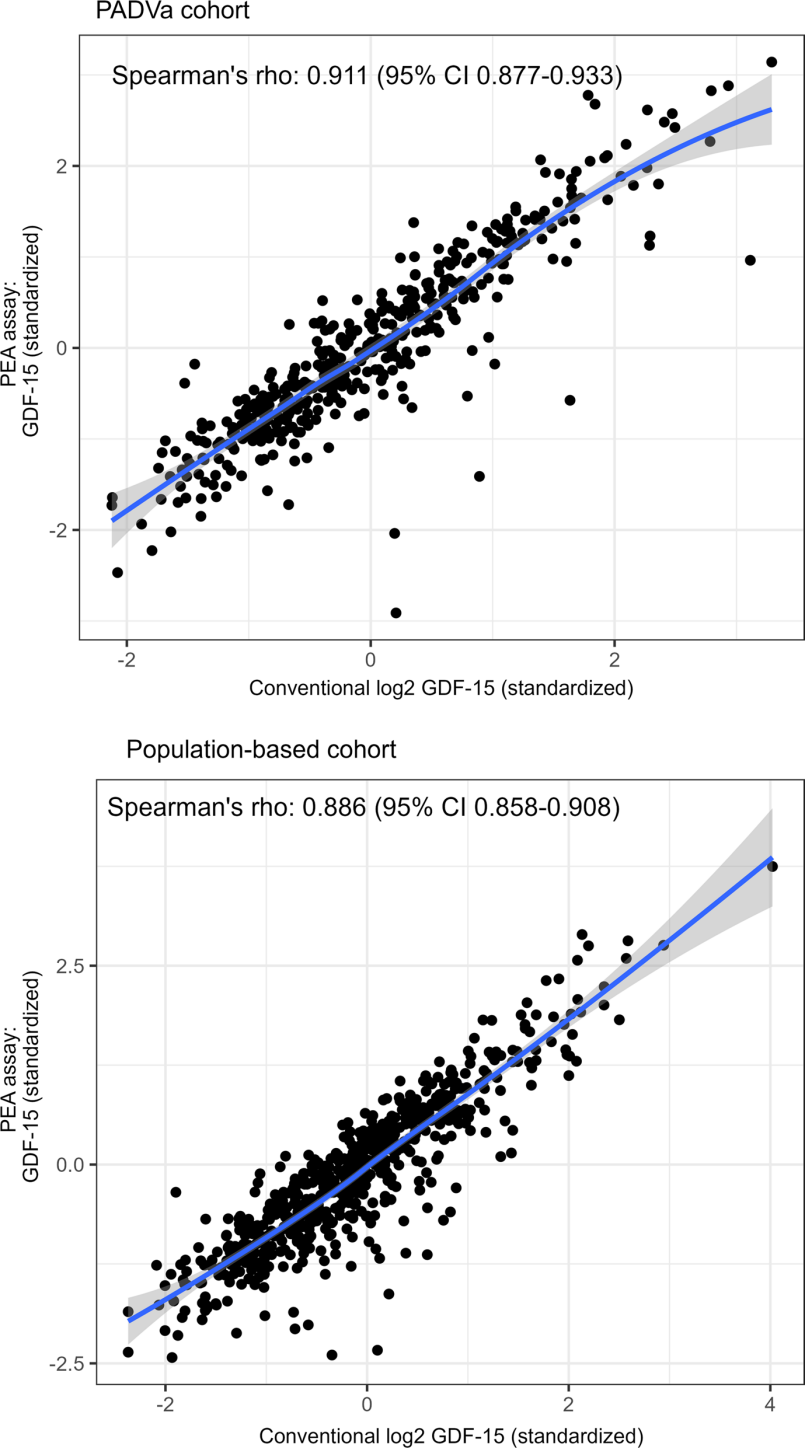
Scatter plots with corresponding curves constructed by LOESS (blue lines) for GDF-15 analyzed with a conventional assay and a PEA assay in the studied cohorts. *LOESS* local polynominal regression lines, *GDF-15* growth differentiation factor 15, *PEA* proximity extension assay, *Spearman´s rho* Spearman’s rank correlation coefficient, *CI* confidence interval

The concentrations of GDF-15 obtained by PEA and conventional technique, respectively, correlated with a coefficient of 0.911 (95% CI 0.877–0.933) in the PADVa cohort and 0.886 (95% CI 0.858–0.908) in the population-based cohort. The association is visualized in a scatter plot (Fig. 2). In contrast to NT-proBNP, no noticeable leveling off in the highest NPX values was seen for GDF-15.

### Prognostic impact of NT-proBNP and GDF-15 from the two analysis techniques

The median follow-up time in the PADVa cohort was 5.2 years during which 98 had an CV event, corresponding to an incident rate of 4.6 events per 100 person-years. In the population-based cohort the median follow-up time was 4.8 years where 34 had an CV event, corresponding 1.2 evens per 100 person-years.

**Fig. 3:**
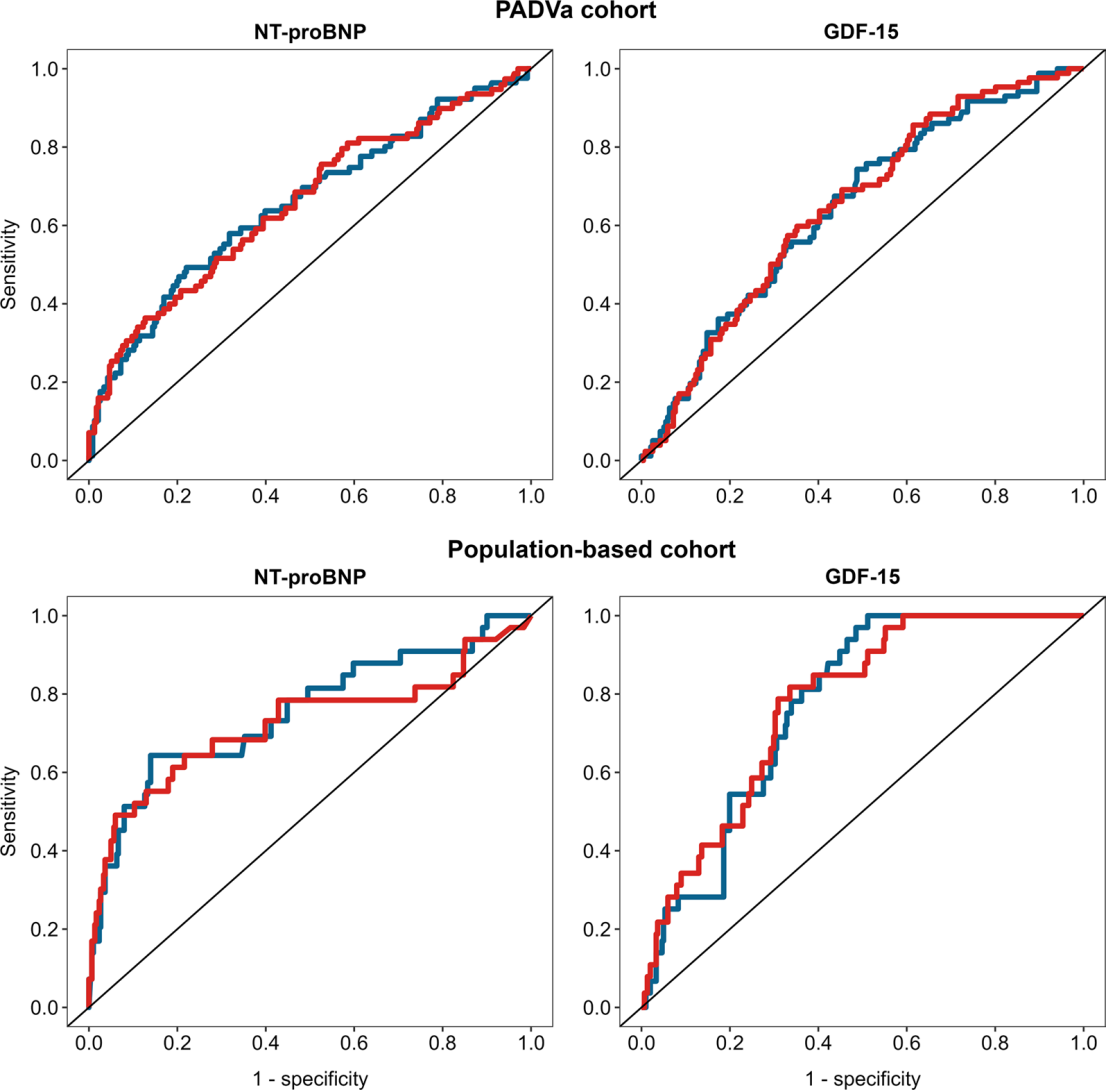
Comparison of time dependent areas under the receiver operating characteristics curve for prediction future CV events for NT-proBNP and GDF-15 measured by different analysis techniques in two different cohorts. The red curve is measured by proximity extension assay and the blue curve is measured by conventional immunoassay. *CV* cardiovascular, *NT-proBNP* N-terminal pro-brain natriuretic peptide, *GDF-15* Growth differentiation factor 15

The hazard ratios were very similar between the conventional and the PEA analyses for NT-proBNP and GDF-15 (Table 2). The discriminatory accuracys, demonstrated by the tdAUCs after 5 years of follow-up, were similar for each protein regardless of which assay technique produced the data. Figure 3 demonstrates the tdAUC for each biomarker by the two assay techniques in both cohorts. In the PADVa cohort, the tdAUC for NT-proBNP was 0.657 (95% CI 0.585–0.729) for the PEA assay and 0.657 (95% CI 0.583–0.730) for the conventional assay. Corresponding figures for NT-proBNP in the population-based cohort were 0.723 (95% CI 0.605–0.841) for the PEA assay and 0.754 (95% CI 0.650–0.858) for the conventional assay.

**Table 2:** Unadjusted Cox regression analysis showing the hazard ratios per one standard deviation increase for NT-proBNP and GDF-15 in predicting CV events measured by Proximity extension assay (PEA) and conventional immunoassay (CIA) in two cohorts

	HR (95% CI)	P-value
PADVa cohort		
NT-proBNP (*PEA*)	1.67 (1.33–2.13)	< 0.001
NT-proBNP (*CIA*)	1.74 (1.44–2.10)	< 0.001
GDF-15 (*PEA*)	1.59 (1.32–1.91)	< 0.001
GDF-15 (*CIA*)	1.56 (1.30–1.87)	< 0.001
Population-based cohort		
NT-proBNP (*PEA*)	2.52 (1.74–3.65)	< 0.001
NT-proBNP (*CIA*)	2.62 (1.96–3.51)	< 0.001
GDF-15 (*PEA*)	2.32 (1.70–3.17)	< 0.001
GDF-15 (*CIA*)	2.26 (1.64–3.12)	< 0.001

*NT-proBNP* N-terminal pro-brain natriuretic peptide, *GDF-15* Growth differentiation factor 15, *CV* cardiovascular, *HR* Hazard ratio per unit change, *CI* Confidence interval, *NPX* Normalized Protein Expression

The tdAUC for GDF-15 in the PADVa cohort was 0.648 (95% CI 0.582–0.715) for the PEA assay and 0.655 (95% CI 0.577–0.712) for the conventional assay. In the population-based cohort the corresponding tdAUCs were 0.774 (95% CI 0.704–0.843) and 0.769 (95% CI 0.705–0.833), respectively.

## Discussion

We found that the relative plasma levels of GDF-15 measured with the PEA assay correlated well with the absolute plasma concentrations obtained with a conventional immunoassay in two parallel cohorts. There was also a strong correlation between the examined assays of NT-proBNP, except for plasma levels above approximately 1500 ng/L. In both cohorts, the prognostic impact of the proteins was similar regardless of the measuring techniques. Our findings suggest that data from the PEA and the conventional immunoassay techniques studied are comparable for these proteins.

The multiplex PEA is a relatively new high-throughput technique increasingly used in human biomarker research. Many promising prognostic biomarkers have been detected using the PEA technique [15, 16, 26, 27]. However, the PEA data are expressed in relative NPX-unit whereas conventional immunoassays present absolute concentrations. Even though data from PEA and conventional immunoassay are presented in different units, comparisons between these data are underexplored. In a small pilot study comprising 120 blood samples from 30 hemodialysis patients, Arrigo et al. demonstrated a Spearman rho of 0.865 between the PEA and a conventional assay for the biomarker brain natriuretic peptide BNP [10]. In our study, including more participants and other proteins, we found strong correlations between values of the PEA and the conventional assays.

The strong correlation between PEA and conventional assays was valid for both NT-proBNP and GDF15 and was similar in both cohorts. However, in both cohorts, the highest NT-proBNP values obtained from PEA leveled off. This can be explained by the hook phenomenon previously recognized as the result of the deteriorating efficiency of antibodies to form an immune complex when the concentrations of an antibody or an antigen are very high [28, 29]. However, this phenomenon though was not observed for GDF-15. The plasma for GDF-15 has, unlike NT-proBNP, been frozen before analysis which may have affected the results.

The highest NT-proBNP values with our PEA panel (Multiplex CVD I 96 × 96) can give inaccurately low NPX-values. Later released PEA panels (Multiplex CVD II and CVD III) include a correction for this deviation with a higher hook limit (http://www.olink.com).

A wealth of data confirms that plasma levels of NT-proBNP and GDF-15 have strong associations with future CV events [30–32]. We aimed to identify the prognostic impact of the two biomarkers and compare the outcome between data obtained by the PEA and conventional assays. This motivated our design with one cohort including individuals with verified PAD, with an expected high incident of late CV events, and one population-based cohort were individuals with PAD were excluded, thus expecting a lower incident of late CV events.

Given the high correlation between the plasma levels obtained by the two different analytical techniques, it was not surprising that the prognostic performance of NT-proBNP and GDF-15 were very similar regardless of the type of assay.

### Strengths and limitations

The similar correlations found between PEA and conventional immunoassay data in two parallel, relatively large cohorts with different CV comorbities strengthens the credibility of our results.

The study was limited to two plasma proteins in the PEA assay, NT-proBNP and GDF-15, and can not be generalized to other plasma proteins. Nor is our results automatically generalizable for other conventional assays from other suppliers. Further studies with more proteins and assays from other suppliers are therefore necessary to conclude a generalized good correlation between PEA and conventional immunoassay data.

Our study used the only Olink cardiovascular Multiplex CVD I panel available at the start of the study. Newer panels have been released. Thus, our results can not be generalized beyond the Multiplex CVD I panel. Further studies on these newer panels are recommended.

The blood samples were stored frozen (− 70 degrees Celcius) for up to 9 years before the PEA analyze. We can not rule out a possible effect on the stability of the proteins then measured. However, the high correlations between the PEA and immunoassay results make such a systematic error less probable.

## Conclusion

We demonstrated a strong correlation and a similar prognostic performance of plasma levels of NT-proBNP and GDF-15 obtained by the PEA assay compared with conventional assays. With the exception of higher levels of NT-proBNP, the findings suggest that the PEA assay reliably reflects plasma levels obtained from conventional assays.

## Data Availability

The dataset used and analysed during the current study are available from the corresponding author on reasonable request.

## Abbreviations

PEA: Proximity extension assay
NT-proBNP: N-terminal pro-brain natriuretic peptide
GDF-15: Growth differentiation factor 15
CV: Cardiovascular
PAD: Peripheral arterial disease
PADVa: Peripheral Artery Disease in Västmanland study
VaMIS: Västmanland myocardial infarction study
ABI: Ankle-brachial index
ICA: Internal carotid artery
LVEF: Left ventricular ejection fraction
CVa: Coefficient of variation
ECL: Elecsys electrochemiluminescence
PCR: Polymerase chain reaction
NPX: Protein expression
MI: Myocardial infarction
SD: Standard deviation
rho: Correlation coefficients
CI: Confidence intervals
LOESS: Polynomial regression lines
tdAUC: Time dependent areas under the receiver operating characteristics curves
HR: Hazard ratio per unit change
CIA: Conventional immunoassay
